# Virtual bolus for total body irradiation treated with helical tomotherapy

**DOI:** 10.1120/jacmp.v16i6.5580

**Published:** 2015-11-08

**Authors:** Gilles Moliner, Françoise Izar, Régis Ferrand, Manuel Bardies, Soléakhéna Ken, Luc Simon

**Affiliations:** ^1^ Departement of Medical Physics Institut Universitaire du Cancer de Toulouse Toulouse France; ^2^ Departement of Radiation Oncology Institut Universitaire du Cancer de Toulouse Toulouse France; ^3^ UMR 1037 Team 15 INSERM Toulouse France

**Keywords:** radiotherapy, TBI, virtual bolus, tomotherapy, fluence peak

## Abstract

Intensity‐modulated radiation therapy (IMRT) for total body irradiation (TBI) is practiced in several centers using the TomoTherapy System. In this context the planning target volume (PTV) is the entire body including the skin. A safety margin in the air surrounding the body should be added to take into account setup errors. But using inverse planning, over‐fluence peak could be generated in the skin region to insure dose homogeneity. This work proposes to study the performance of the use of a virtual bolus (VB). A VB is a material placed on the skin surface during planning, but absent for the real treatment. The optimal VB that compensates large setup errors without introducing a high‐dose increase or hot spots for small setup errors was determined. For two cylindrical phantoms, 20 VBs with different densities, thicknesses or designs were tested. Dose coverage of the PTV (V95%) in the presence of simulated setup errors was computed to assess the VB performance. A measure of the dose increase in the phantom center due to the absence of the VB during treatment was also achieved. Finally, the fluence peak at the phantom edge was measured in complete buildup conditions using a large phantom and a detector matrix. Using these VBs, simulated setup errors were compensated to a minimum value of 2.6 and 2.1 cm for small and large phantom, respectively (and only 1.2 and 1.7 cm with no VB). An optimal double‐layer VB was found with a density of $0.4\;\text{kg}.m^{-3}$ and a total thickness of 8 mm; an inner layer of 5 mm was declared as the target for the treatment planning system and an additional layer of 3 mm was added to avoid the over‐fluence peak. Using this VB, setup errors were compensated up to 2.9 cm. The dose increase was measured to be only +1.5% at the phantom center and over‐fluence peak was strongly decreased.

PACS numbers: 87.53 Bn, 87.55 D‐, 87.55 de, 87.55 dk

## I. INTRODUCTION

Total body irradiation (TBI) has been conventionally used with chemotherapy as preparatory regime of bone marrow transplantation (BMT) since 1959.^(1)^ It allows immunosuppression to decrease the risk of graft‐versus‐host disease (GVHD) and also kills malignant cells to help acceptance of donor transplant.^(2)^ Today, TBI is mainly achieved with conventional radiotherapy (RT) using anteroposterior fields and a long source–skin distance (SSD). Limits of this 50‐year old technique have been previously described: dose heterogeneity, long treatment time, low ability to protect organs at risk (OAR) and, thus, acute and late toxicity.^3^, ^4^, ^5^, ^6^, ^7^, ^8^, ^9^, ^10^, ^11^, ^12^ Moreover, treatment planning systems (TPS) are not useable with this technique. No plan is computed before the treatment, so knowledge about the dose distribution is limited. This treatment is usually driven using *in vivo* detectors measurements (e.g., diodes, TLDs). This makes it possible to evaluate the dose only in a few points of the patient. Finally, the dose is prescribed at a point (i.e., abdomen) and assessed at some other points of the body (e.g., head, legs). Lungs are sometimes protected with shielding blocks.

Recently, some patients were treated using Helical Tomotherapy (HT) (Accuray, Sunnyvale, CA).^13^, ^14^, ^15^, ^16^ Intensity‐modulated RT (IMRT), like HT, is highly conformal and, using inverse planning methods, allows the treatment of a complexly shaped volume while sparing neighboring tissues. A setup margin is generally used to create the planning target volume (PTV).^(17)^ But when the PTV is close to, or even outside, the body surface (i.e., in TBI case), it is common to crop it 2 or 3 mm under the skin. Indeed, to insure the dose homogeneity in the PTV, inverse planning TPS strongly increases the photon fluence in these regions with low electronic buildup. This creates an over‐fluence peak and could lead to hot spots in the case of small setup errors.

In the context of TBI, the target is the whole body. Large setup errors (>1 cm) are likely, even with a patient immobilization system and pretreatment 3D imaging.^(1)^ This could lead to underdose of some regions. It is not possible on the TomoTherapy planning station to modify manually the beam aperture for treatment of the flash region, as recommended in ICRU report 83.^(18)^ The use of "pretend" or "virtual" bolus (VB) in TomoTherapy planning has been proposed by several authors.^13^, ^16^, ^19^ VB consists of adding an artificial material (a bolus with chosen density and thickness) on the skin during planning, which is then removed for treatment. VB is declared as the "target" for the optimization algorithm. In this context of inverse planning, a "target" structure is one that the algorithm will try to cover with a minimum dose (whereas an OAR is a structure for which the algorithm tries to limit the dose). Thus it generates a multileaf collimator (MLC) sequence that takes into account the setup margin. But it also slightly increases the delivered dose, because patient thickness, from the TPS point of view, is larger than the real thickness. This study proposes to determine the optimal density and thickness of a VB to treat TBI using HT. Using two cylindrical phantoms (small and large), HT plans with several VB thicknesses and densities (including no VB) were achieved. In the first part, the ability of these VB to compensate simulated setup errors was assessed. Then, in the second part, the modification of the dose distribution induced by the use of VB was studied. The increase of the dose in the central region, as well as the distribution just under the skin surface, were both observed. According to these two studies, the optimal VB that allowed compensating setup errors while keeping acceptable the dose distribution was determined.

## II. MATERIALS AND METHODS

### A. Images and contours

Two cylindrical phantoms were used for this study: the cheese phantom (Accuray Inc., Sunnyvale, CA), hereafter called thorax phantom, and a "CTDI" phantom usually dedicated to CT dose measurement, hereafter called leg phantom. Diameters of thorax and leg phantom were 30 and 16 cm, respectively, and they were considered as equivalent to water. CT scans were acquired for both phantoms (slice thickness equal to 2.5 mm). CT images were imported to Eclipse 11.0 (Varian Medical System, Palo Alto, CA) for contouring. For both phantoms, the following contours were created: body (external contour of the whole phantom), PTV (body – 3 mm limited in superior–inferior (SI) direction to a central region of 8 cm), and several other VB around the body, as follows. VB were drawn as external rings all around the body starting from the skin surface and with different thicknesses. VB were limited in SI direction to the PTV region because this study was limited to axial setup errors (in the gantry plane). Four different VB were tested: two single‐layer VB of 5 and 10 mm thickness and two double‐layer VB of 5+3 and 10+3 mm thickness. Single‐layer VB is totally declared as target to the optimization algorithm. For double‐layer VB, the inner layer is declared as target, but not the external additional 3 mm. Compared to a single‐layer VB, the use of this double‐layer VB design could avoid the over‐fluence peak previously described. Single‐layer VB is often reported in the literature as pretend bolus (density is generally equal to surrounding tissue). Single‐layer VB in our study is slightly different from "skin flash method", which involves enlarging the MLC aperture after the optimization process (in this case, density is zero).

**Figure 1:**
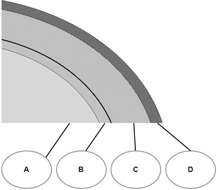
Schematic view of the VB designs: A = inner part of the phantom; B = external layer of the phantom (3 mm); C = inner part of VB (5 or 10 mm); D = external part (3 mm) of the VB. A and C were declared as targets to the TPS. B and D were not declared as targets. D is only present for double‐layer VB. Four designs were tested: two single‐layer $\left({\text{VB}}_{5},{\text{VB}}_{10}\right)$ and two double‐layer $\left({\text{VB}}_{5+3},{\text{VB}}_{10+3}\right)$. The first number refers to the thickness of C and the second number refers to the thickness of D.

Hereafter, these single‐ and double‐layer designs will be denoted as ${\text{VB}}_{x,\rho =d}$ and ${\text{VB}}_{x+y,\rho =d}$, respectively, where *d* is the VB density $\left(\text{kg}.m^{-3}\right)$ and *x* and *y* are the layer thicknesses. Schematic VB design is presented in Fig. 1.

### B. Initial plans

Images and contours were exported to TomoTherapy planning station (PS) v2.0.1 for planning using the Volo algorithm. All TomoTherapy plans were computed with the same parameters: pitch, dynamic collimation, and modulation factor were equal to 0.43, 5 cm, and 2.0, respectively. For reproducibility, only 100 iterations were performed without modifying the objectives. The PTV and one of the VB were declared as targets with respective optimization importance of 10 and 1. The prescription was 12 Gy (2 Gy/fraction) to the median volume of PTV. VB density was set successively to one of the five following values: 0, 0.1, 0.2, 0.4 or 1 $\text{kg}.m^{-3}$. For each density value, thickness was set to the 5, 10, 5+3, and 10+3 mm (see previous section). In total, 21 initial plans were computed for both phantoms (i.e., 42 plans), including 20 plans with a VB and one plan with no VB (i.e.,thickness=0).

### C. Simulated setup errors

**Figure 2:**
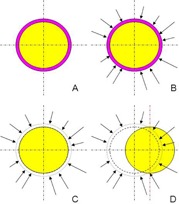
Study of VB performances to compensate setup errors. The whole phantom (light grey area) and a VB (dark grey area) are defined as targets (a). A modulated plan is computed (b). Fluence of this plan is used to compute the dose distribution in the same phantom but without the VB (c). The same computation is performed after the introduction of different setup errors (d). Coverage of the phantom is assessed using the phantom DVH.

VB should insure dosimetric coverage in case of setup errors. To simulate these errors and assess VB performances, the helical fluence of the initial plans was used and the dose was computed in the phantoms after the removal of the VB. A shift along the x‐axis was introduced (Fig. 2). DQA station (Accuray Inc.) was used to compute, for each of the 42 initial plans, four quality assurance (QA) shifted plans using a x‐shift of 0, 1, 2, and 3 cm (total 168 QA plans). These shifts (up to 3 cm) can be considered as very large, but in the context of TBI, some large and significant setup errors can be expected. QA plans with no shift (0 cm) made it possible to assess the underestimation of dose induced by the VB methodology (fluence was optimized with a VB, treatment was delivered without). This assessment was confirmed by measurement (see next section).

A MATLAB script (MathWorks, Natick, MA) was used to edit the DICOM header of CT images to allow the same CT series to be used both as a "patient" and as a "phantom" in TomoTherapy PS (something which is not normally possible). RT dose files of initial and QA plans were imported into Eclipse to assess the dosimetric figures of merit for PTV: V95% (volume percentage of the PTV receiving 11.4 Gy), and Dmax (maximum dose).

For each of the 42 initial plans, the lateral Maximum Acceptable Set‐up Error (MASE) was determined — the setup error (cm) for which 95% of the PTV still received 95% of the dose. If the setup error was greater than the MASE, the target was not considered as correctly treated.

### D. Underestimation of the dose (measurement and computation)

In presence of VB during planning, the total body thickness for computation is greater than the real body thickness. Thus, a global underestimation of the delivered dose to the body was foreseen. To study this effect, the following measurements were taken: all previously described plans were delivered to both phantoms (leg and thorax). An absolute measurement was achieved with an ionization chamber (A1SL, Standard Imaging, Middleton, WI) located at the center of the cylinders. The underestimation of the dose was assessed by observing the dose increase relative to the plan with no bolus: $(D_{\text{VB}}-D_{\text{NB}})/D_{\text{NB}}$, where $D_{\text{VB}}$ and $D_{\text{NB}}$ are the doses with and without VB, respectively. For this estimation of the dose increase, two additional VB densities (0.6 and 0.8 $\text{kg}.m^{-3}$) were assessed.

This dose increase measurement was compared to the TPS results (previous section). $D_{\text{VB}}$ and $D_{\text{NB}}$ were obtained from the planning station when looking for the center dose on initial plans and nonshifted QA plans, respectively (which are copies of the initial plans without bolus).

### E. Over‐fluence peak

**Figure 3:**
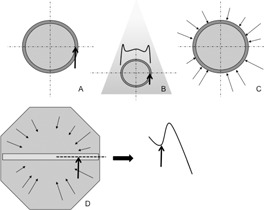
Study of the over fluence peak. A plan is computed using a VB and modulated fluence ((a) and (c), see Fig. 2). An example of a possible fluence for one beam direction is shown on (b); high fluence is needed to give a homogeneous dose to the low buildup regions at the phantom edges. The plan is delivered to a larger phantom (OCTAVIUS phantom) including a detector matrix (d). This insures a complete electronic buildup. Dose profile (dashed line) is measured and the over‐fluence peak (in the VB region) is estimated relative to the "no bolus" plan. The vertical arrow represents the edge of the initial phantom.

To deliver a homogeneous dose to regions with low buildup (i.e., low‐density regions and the first few millimeters under the skin), optimization algorithms increase the photon fluence. For very low densities, an over‐fluence peak can be observed. This over‐fluence peak is not recommended, because it could lead to skin overdosage in the case of small setup errors. To study this effect for VB, the previously described initial plans were delivered to the OCTAVIUS phantom (PTW, Freiburg, Germany), including a detector matrix (Matrix 729, PTW). The OCTAVIUS phantom was located such that the over‐fluence peak could be observed in conditions of complete electronic buildup (Fig. 3). A central profile (in the X direction) was extracted from the 2D acquisition. For all VB, these profiles were compared to the profile obtained for the plan without VB. This work was completed only for the leg phantom; the fluence peak was assumed to be independent of phantom diameter.

## III. RESULTS

### A. Simulated setup errors

**Figure 4:**
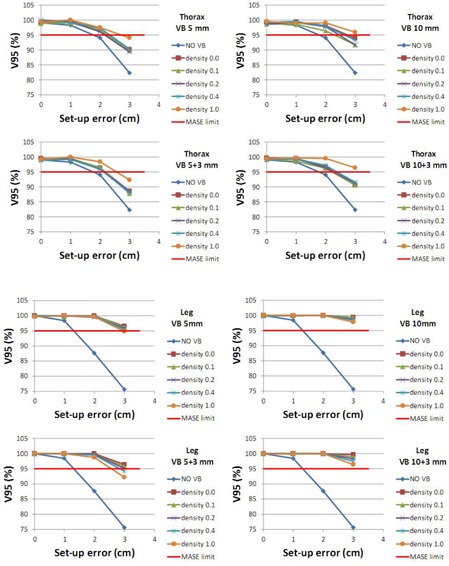
V95% (relative PTV volume receiving 95% of the prescribed dose) in the presence of simulated setup errors. Results are shown for thorax (top four charts) and leg (bottom four charts) phantoms and for VB thickness equal to 5, 5+3, 10, and 10+3 mm. No VB=No Virtual Bolus. The red horizontal line represents the MASE limit. Under this line, V95% is lower than 95% and PTV coverage is not sufficient.

**Figure 5:**
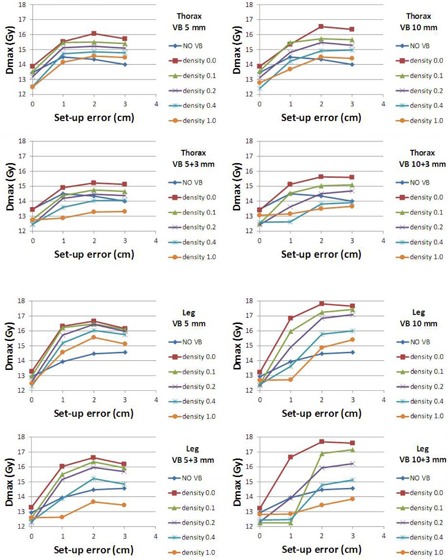
Maximum dose (Gy) in the PTV for different densities $\left(\text{kg}.m^{-3}\right)$ of the Virtual Bolus and for simulated setup errors (cm). Results are shown for thorax (top four charts) and leg (bottom four charts) phantoms and for VB thickness equal to 5, 5+3, 10, and 10+3 mm.No VB=No virtual bolus.

V95% and Dmax are presented in Fig. 4 and Fig. 5, respectively, for both thorax and leg phantoms. These figures show the decrease of V95% and increase of Dmax when the lateral setup errors increase for different densities and thickness of VB. In Fig. 4, MASE is assessed as the maximum setup error that still insures a good PTV coverage (V95>95%).

### B. Underestimation of the dose (measurement and computation)

**Figure 6:**
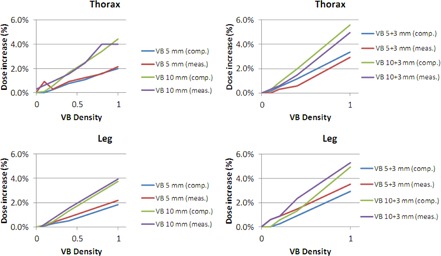
Measure (meas.) and computation (comp.) of dose increase at the phantom center relatively to the plan with no bolus. Results are presented for thorax (top) and leg (bottom) phantoms, for different VB thickness and densities ($\text{kg}.m^{-3}$).

Figure 6 shows the dose increase at the phantom center when using a VB, relative to a plan without a VB. This dose increase can be understood as the error made on dose computation when using the VB methodology (computation with, and treatment without the VB material). Figure 6 shows the measurements with the ion chamber of this dose increase, as well as the same ratio obtained from computation on the TPS (previous section).

### C. Over‐fluence peak

**Figure 7:**
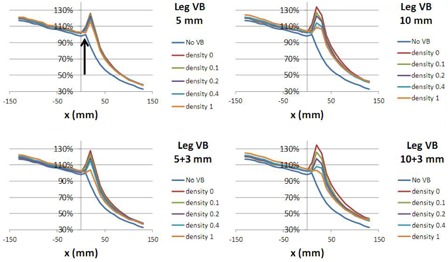
Over‐fluence peak observed at the edge of the phantom measured in buildup conditions (see Fig. 3). All graphs must be interpreted as relative measurement and compared to the plan with no virtual bolus (No VB). All figures are arbitrarily normalized to the phantom edge of the plan with no bolus (arrow). The left part of each curve corresponds to the inside of the actual phantom (under the VB). The x‐axis is in mm and corresponds to the left–right direction of the gantry plane. The x‐axis origin is arbitrary. For the plans "No VB" and double‐layer VB, PTV is clipped 3 mm below the surface (see text).

Figure 7 shows the x‐axis profiles extracted from the 2D acquisitions to assess over‐fluence peak for different VB thicknesses and densities. These measurements were obtained in complete buildup conditions. The profile for the plan without VB was also represented and used as a reference (PTV is drawn 3 mm under the phantom surface for this plan).

## IV. DISCUSSION

This work has been undertaken to find the optimal VB that insures a good coverage (V95%>95%) of the total body during TBI in presence of possible setup errors (more than 1 cm). The following discussion details the reasons why an optimal VB with a thickness of 5+3 mm and a density of $0.4\;\text{kg}.m^{-3}$ was selected. To choose this optimal VB $\left({\text{VB}}_{5+3,\rho =0.4}\right)$ the following issues were considered.

Table 1 summarizes all the results for the leg phantom and should help the reader to follow the discussion.

**Table 1:** Results for leg phantom. MASE is interpolated or extrapolated using a linear fit from Fig. 4

		*Bolus Thickness (mm)*				
	*Density*	*No VB*	*5*	5+3	*10*	10+3
MASE (mm)	0	13	31	34	>35	>35
	0.1		33	32	>35	>35
	0.2		32	30	>35	>35
	0.4		31	29	>35	>35
	1		33	26	>35	>35
Dmax (Gy) (setup error=1 cm)	0	13,94	16,30	16,03	16,84	16,64
	0.1		16,21	15,51	15,96	12,24
	0.2		15,71	15,15	14,89	13,90
	0.4		15,19	13,87	13,63	12,46
	1		14,55	12,61	12,72	12,84
Dose increase (%) vs. no bolus (measurement)	0	‐	−0,1%	0,0%	−0,2%	0,0%
	0.1		0,1%	0,6%	0,3%	0,6%
	0.2		0,3%	0,9%	0,7%	0,9%
	0.4		0,8%	1,5%	1,6%	2,4%
	0.6		1,3%	2,2%	2,4%	3,4%
	0.8		1,8%	2,9%	3,2%	4,4%
	1		2,2%	3,5%	3,9%	5,3%
Over‐fluence peak (100%=phantom edge with no bolus)	0	117%	126%	127%	134%	134%
	0.1		124%	123%	127%	126%
	0.2		122%	119%	123%	121%
	0.4		121%	120%	121%	122%
	1		121%	122%	124%	125%

### A. Dose coverage in case of setup errors

Figure 4 shows that with no VB (curve None), the lateral MASE (maximum error for which 95% of PTV still receiving 95% of the dose) is 1.2 cm and 1.7 cm, for leg and thorax phantom, respectively. Regardless of the VB used, these values are at least equal to 2.6 cm and 2.1 cm.

Considering these results, the VB is assumed to be more necessary for smaller parts of the patient's body (smaller MASE without VB) and also more effective (greater MASE benefit when using VB). Moreover, for the leg phantom, if the error is greater than the MASE, the curve falls off quickly. This means that for large setup errors, the small parts of the patient (legs) are particularly underdosed. In our institute, setup errors for TBI are more important for legs than for the rest of the body (a thermoformable mask is achieved for head, thorax, and abdomen, but not for legs). According to these first results, it was decided to use VB only on patient legs (and not on head, thorax, and pelvis).

Obviously the VB thickness has an influence on the MASE increase for both phantoms. The thicker the VB is, the greater the MASE is. For a large 3 cm setup error, for thorax phantom with ${\text{VB}}_{5,\rho =1}$ and ${\text{VB}}_{10,\rho =1}$, V95% was 94.0% and 95.9%, respectively, (and only 82.4% if no VB is used). This effect is even bigger for leg phantom, for which these values are 94.9% and 97.9% (and 75.6% with no VB).

Density of the VB has a moderate influence on V95%. In Fig. 4, there is a large difference between the no‐bolus curve and all other VB curves. However, all the VB curves are very similar. This can be explained because, if a VB is declared as a PTV to the optimization algorithm, it creates an MLC sequence, more widely opened than when no bolus is used, regardless of the density value. Thus, a larger volume is irradiated around the target and then, setup error is better compensated.

The chosen VB $\left({\text{VB}}_{5+3,\rho =0.4}\right)$ had a MASE of approximately 2.9 cm for leg phantom.

### B. Maximum dose

On the other hand, VB density has a large influence on the maximum dose, in particular in the presence of setup errors (see Fig. 5). As an example, the following value can be highlighted. In leg phantom case, with no bolus and no setup error, Dmax was 107.7% (12.92 Gy). When a 1 cm setup error is simulated, this value becomes 116.2%. If a low‐density VB is used (i.e., ${\text{VB}}_{10,\rho =0}$) with the same 1 cm setup error, Dmax becomes 140.3%. This value represents an important overdosage. When a higher density is used, Dmax becomes more reasonable: for ${\text{VB}}_{10,\rho =0.4}$ and ${\text{VB}}_{10,\rho =1}$, it was 113.6% and 106.0%, respectively. The chosen VB (${\text{VB}}_{5+3,\rho =0.4}$) had an acceptable Dmax of 115.5%.

### C. Underestimation of delivered dose in presence of VB

Obviously, the density of VB must not be too high. Indeed, when density (or thickness) increases, it also increases the computation error (difference between the plan and the real treatment). Figure 6 shows that the computation with a VB induced an underestimation of the delivered dose at the phantom center that ranges from 0.0% to 5.2% (maximum for ${\text{VB}}_{10,\rho =1}$). According to our physicians, a 3% error in this clinical context can reasonably be accepted, in particular compared to the classic TBI technique.^(18)^ The use of the chosen VB (${\text{VB}}_{5+3,\rho =0.4}$) leads to an underestimation of 1.5%. In this figure, it is noticeable that the dose increase measurement is well described by the TPS (computed and measured curves are very similar).

Good results were obtained for the VB with density 1 $\text{kg}.m^{-3}$ in all other tests presented in this work (e.g., setup errors, Dmax). But it was dismissed from the clinical use due to this excessive increase in dose. Indeed, all the dose distribution (including hot spots and not only central dose) could be increased with this value.

### D. Over‐fluence peak

Usually, when a VB is used, the whole thickness of the additional material is declared as a PTV. This is not consistent with report 83 of ICRU^(19)^ that recommends, for inverse planning, to let the first millimeters of skin remain outside the PTV. This is the reason why double‐layer VB was assessed, with thickness of 8 mm (5+3) and 13 mm (10+3), including 3 mm of buildup not declared as PTV. In Fig. 7, the following points can be observed.

For these two double‐layer VB with a density of 1 $\text{kg}.m^{-3}$, the dose profile shape with a complete buildup was the same than with no VB. This result was expected because, for no VB plans, the PTV was also drawn 3 mm under the skin (see Fig. 1).

On the contrary, when the density is too low (i.e., 0 $\text{kg}.m^{-3}$), there is a large over‐fluence peak located in the VB region. Again, to deliver a dose to a low‐density region, the optimization algorithm creates a very high photon fluence. This is the reason why a very high Dmax, in presence of small setup errors (Fig. 5), is observed.

Relative to the case with no VB, maximum value of the fluence peak was 115% for the chosen VB (${\text{VB}}_{5+3,\rho =0.4}$). This value was 119% for the same VB without the additional 3 millimeters (${\text{VB}}_{5,\rho =0.4}$).

${\text{VB}}_{5+3,\rho =0.4}$ was preferred to ${\text{VB}}_{5,\rho =0.4}$ for this reason. Indeed, this 4% difference can be linked to the behavior of Dmax in Fig. 5. In presence of 1 cm setup error, for ${\text{VB}}_{5+3,\rho =0.4}$ and ${\text{VB}}_{5,\rho =0.4}$, Dmax was 115% and 127%, respectively.

### E. General discussion

These previous results regarding setup error compensation and over‐fluence peak limitation are acceptable in the clinical context of TBI. But this study could be useful for other treatments. Chest wall, breast, and head‐and‐neck patients are treated with Helical Tomotherapy in our department. For these patients, it is very common to observe PTV close to the skin. A VB could be useful to achieve dose coverage of the skin region, even in presence of setup errors, without introducing hot spots. This original methodology can be an alternative to the solution proposed in Report 83 of the ICRU: the flash region method that consists in manual beam apertures and manual intensity definitions.^(19)^

As a future improvement, it could be of interest to assess the performances of VB with different densities for the inner and the outer layer. A higher density for the inner layer would limit the over‐fluence peak in the skin region, and a lower density for the outer layer would limit the dose computation error.

Since summer 2014, seven patients were treated using Helical Tomotherapy and a VB in our department. Six fractions of 2 Gy were delivered (2 fractions per day during three days) to the entire body (except the lungs, which received a mean dose of 8 Gy). During planning, the chosen VB (${\text{VB}}_{5+3,\rho =0.4}$) was drawn all around the entire legs (from feet to approximately the mid‐height of thighs). The plan was optimized with VB and presented to the physician. A nonshifted QA plan (dose delivered when the fluence of the VB plan is delivered to the patient without VB, as previously described) was also presented to the physician during validation process, to assess the dose actually delivered to the patient (excluding the dose underestimation).

Some teams reported the use of a real bolus for ribs or hands because bones are close to the skin surface in this region. This solution was not adopted in our department because of the lack of reproducibility of bolus position between CT and treatment.

## V. CONCLUSIONS

For TBI treatment, several types of VB were tested to find the optimal model that gives the best compromise between dose coverage of the target in presence of setup errors and global dose increase. In the first part it was decided to use a VB only for patient legs. Indeed, VB is more necessary and also more effective for the legs (small phantom) than for thorax (big phantom). A VB with density $0.4\;\text{kg}.m^{-3}$ and 8 mm thickness (5 mm plus 3 mm excluded of PTV) was finally chosen. This VB (${\text{VB}}_{5+3,\rho =0.4}$) insures good dose coverage (V95%>95%) for setup errors of up to 2.9 cm (MASE). With no VB, MASE was 1.2 cm. In the presence of this chosen VB, Dmax for a 1 cm setup error is 115%. This value was kept reasonable because the 3 first mm are excluded of PTV. This methodology strongly decreases the over‐fluence peak. The general dose increase (underestimation of dose due to the presence of VB during computation) was measured at the phantom center and was equal to 1.5%.

## ACKNOWLEDGMENTS

The authors declare no conflict of interest. We would like to thank David Schaal and Mikail Gezginci for proofreading the manuscript.
